# The effects of gastrointestinal disturbances on the onset of depression and anxiety

**DOI:** 10.1371/journal.pone.0262712

**Published:** 2022-01-25

**Authors:** David Cantarero-Prieto, Patricia Moreno-Mencia

**Affiliations:** 1 Group of Health Economics and Health Services Management-IDIVAL, Santander, Cantabria, Spain; 2 Department of Economy, University of Cantabria, Santander, Spain; 3 Department of Economy, International University of La Rioja, Logroño, Spain; Shanghai Jiao Tong University, CHINA

## Abstract

**Background:**

Stomach pain is an ailment that occurs frequently in the general population. It is not unusual if such stomach issues produce some amount of stress in an individual, but it can be worrying if these pains lead to significant mental health problems. The relationship between some abdominal pain, such as bowel syndrome, and depression or anxiety has been gaining much interest. However, previous studies that have empirically investigated this relationship are scarce.

**Methods:**

To analyze the impact of having gastrointestinal problems, among other socioeconomic conditions, on the onset of depression and anxiety in the Spanish population, we compare treating gastrointestinal diseases as exogenous in a single-equation probit model with a bivariate probit model in which this variable is treated as endogenous. A likelihood-ratio test of the correlation coefficient of the disturbances suggests that gastrointestinal problems are endogenous. Thus, the approach taken herein allows the direct testing of the hypothesis that having gastrointestinal problems and the onset of mental illnesses such as depression and anxiety are jointly determined by certain socioeconomic factors. A cross-sectional analytical study was analyzed using data from a 2017 survey of health indicators and life conditions that was developed by the Statistics Spanish Office.

**Results:**

The probability of having depression or anxiety increases with age, stress, daily limitations and gastrointestinal disorders. On the other hand, the probability is lower for men, married people and those who engage in exercise several times per week. Our findings estimate that having gastrointestinal problems increases the probability of having depression in 7% and the probability of anxiety in 8.8% of the sample.

**Conclusions:**

Our empirical results suggest that not considering the endogeneity of gastrointestinal problems could result in an overestimate of the impact of this factor on the development of depression or anxiety.

## Background

Some evidence exists about the relationship between digestive illnesses and certain mental disorders ([1, 2]). According to health professionals, the brain has a direct effect on our stomach, such that the two are strongly connected. Gastrointestinal diseases are usually composed of chronic diarrhea, nausea, vomiting, irritable bowels or stomach pain, and people suffering these symptoms often experience some psychological problems and stress related to them. In this way, it is not a new idea that chronicity affects mental health; in fact, a large body of studies exists that analyze the association between mental illness and other chronic diseases. The relationship between some abdominal pain, such as bowel syndrome, and depression or anxiety has been gaining much interest ([3–5]). However, few studies have focused on the association between some gastrointestinal problems and the onset of common mental problems, such as anxiety or depression. In this sense, Alander et al. [6] showed that patients with gastrointestinal diseases report having higher levels of stress and psychological problems than healthy patients. Bener et al. [7] found that stress is a strong predictor of the level of severity in patients with diabetes and gastrointestinal disorders. The aim of the current study was to test whether the prevalence of mental disorders such as depression or anxiety effectively differ significantly between patients with gastrointestinal symptoms and those without gastrointestinal symptoms. The econometric framework used herein accounts for the interaction between dummy endogenous variables. The bivariate probit model is a joint model containing two binary dependent variables whose error terms are supposed to be correlated, which matches our proposed analysis. Additionally, we propose to estimate univariate probit regressions for each outcome to see the impact of the chosen regressors. Alternatively, this endogeneity challenge could be managed through Mendelian randomization [8] or analysis a switching regression formulation, similar to that found in previous research ([9, 10]).

In summary, through this first-attempt, it is possible to estimate the correlations observed between these variables by treating the onset of gastrointestinal problems as an exogenous determinant of depression. However, these two diseases may be jointly determined, either by socioeconomic characteristics or because in the population, the probabilities of having depression and gastrointestinal problems are correlated in some way. Moreover, if gastrointestinal variables are endogenous in the depression equation, it is also necessary to address the additional difficulty while estimating the model. Hence, we present a bivariate probit model able to deal with the challenge proposed ([11]). Our paper contributes to the field literature because we consider the heterogeneity in the treatment effect.

## Methods

In this study, we deal with two binary outcomes, namely, the existence of gastrointestinal disturbances and the onset of depression or anxiety. As we have explained in the introduction section, it is reasonable to establish the joint modeling of both outcomes. Let us then define the following:

- *y*_1_ is the first outcome. It is a binary variable indicating whether the person has depression or anxiety.
- *y*_2_ is an endogenous binary variable and the second outcome that takes the value of 1 if the person has a gastrointestinal disease.

### Statistical analysis

Now that we have defined the outcomes of interest and following the popular approach of Heckman (see [12]), we propose the estimation of the endogenous effect using a simultaneous equation system through a bivariate probit model. In this econometric framework, the difficulty is to study the effect of a binary endogenous regressor on another binary outcome variable. Let us define the following:

$$\begin{array}{c} y_{1i}^{*}=x\beta +\alpha y_{2i}+u_{1i} \end{array}$$ (1)

where y* is an unobservable variable. However, we can observe the final outcome of each individual after this latent decision, which is as follows:

$$\begin{array}{c} y_{1i}=0\;\text{if}\;y_{1i}^{*}<0\\ y_{1i}=1\;\text{if}\;y_{1i}^{*}\geq 0 \end{array}$$ (2)

Finally, we observe whether the person actually has depression or anxiety. If the answer is affirmative, then we observe *y*_1*i*_ = 1. Additionally, we assume that the error term has a variance $\sigma_{u}^{2}$, which is usually set to be 1. Moreover, it is possible to specify the treatment equation as follows:

$$\begin{array}{c} y_{2i}^{*}=z\gamma +u_{2i} \end{array}$$ (3)

It is assumed that both error terms, *u*_1_ and *u*_2_, are independent of the explanatory variables (*x* and *z*); however, it is not necessary to assume that these errors are independent between them, which, as we have analyzed, is reasonable. Thus, it is possible to define the second outcome of interest as follows:

$$\begin{array}{c} y_{2i}=0\;\text{if}\;y_{2i}^{*}<0\\ y_{2i}=1\;\text{if}\;y_{2i}^{*}\geq 0 \end{array}$$ (4)

where, if we denote *w* = (*x*, *z*), *θ* = (*β*, *α*) and assume that the function Ψ(⋅) is a cumulative distribution function in the probability measure {0, 1}, then:

$$\begin{array}{ccc} P[y_{j}^{*}>0|w] & = & P[u_{j}>-w\theta |w]\\ =P[u_{j}<w\theta |w] & = & P\left[y_{j}=1|w\right]=\Psi \left(y_{ji}^{*}\right) \end{array}$$ (5)

For each observation, the density of *y* given *w* may be written as follows:

$$\begin{array}{c} f\left(y|w\right)={\left[\Psi (w_{i}\theta )\right]}^{y_{i}}{\left[1-\Psi (w_{i}\theta )\right]}^{1-y_{i}},\;y_{i}=0,1 \end{array}$$ (6)

The log-likelihood for the observation *i* is as follows:

$$\begin{array}{c} \ell_{i}\left(\theta \right)=y_{i}\text{log}[\Psi (w_{i}\theta )]+\left(1-y_{i}\right)\text{log}[1-\Psi (w_{i}\theta )] \end{array}$$ (7)

The parameters of interest are estimated by using maximum likelihood techniques (see Chapter 17 of [11] for more detailed information). In the special case of the probit model, Ψ(⋅) is the *CDF* of the normal distribution function. According to this, it is possible to write the joint distribution of *y*_1_ and *y*_2_ conditional on the explanatory variables as follows:

$$\begin{array}{ccc} P[y_{1}=0,y_{2}=0|x,z] & = & P[u_{1}\leq -x\beta ,u_{2}\leq -z\gamma ] \end{array}$$ (8)

$$\begin{array}{ccc} P[y_{1}=1,y_{2}=0|x,z] & = & P[u_{1}>-x\beta ,u_{2}\leq -z\gamma ] \end{array}$$ (9)

$$\begin{array}{ccc} P[y_{1}=0,y_{2}=1|x,z] & = & P[u_{1}\leq -x\beta -\alpha ,u_{2}>-z\gamma ] \end{array}$$ (10)

$$\begin{array}{ccc} P[y_{1}=1,y_{2}=1|x,z] & = & P[u_{1}>-x\beta -\alpha ,u_{2}>-z\gamma ] \end{array}$$ (11)

It is possible to obtain these four probabilities once the joint distribution of *u*_1_ and *u*_2_ is established. We have assumed that *F*(*u*_1_, *u*_2_) = Φ_2_(*u*_1_, *u*_2_, *ρ*), and in this case, *ρ* is the correlation coefficient. If it is also considered that *E*(*u*_1_) = *E*(*u*_2_) = 0 and *var*(*u*_1_) = *var*(*u*_2_) = 1, then in this case, *ρ* = *cov*(*u*_1_, *u*_2_). Another possibility is to let this joint distribution remain unrestricted by using a semiparametric or nonparametric function, as in Wang et al. [13]. Moreover, the model, $y_{1i}^{*}=x\beta +\alpha y_{2i}+u_{1i}$, can be disaggregated as follows:

$$\begin{array}{c} y_{1i}^{*}=y_{2i}y_{11i}^{*}+\left(1-y_{2i}\right)y_{10i}^{*} \end{array}$$

where

$$\begin{array}{ccc} y_{11i}^{*} & = & x\beta +\alpha y_{2i}+u_{11i},\\ y_{10i}^{*} & = & x\beta +u_{10i} \end{array}$$

Given that nonlinearities exist in the selection mechanism, we do not need exclusion restrictions for model identification. To make our estimates, we have included in vector *z* at least one variable that is not included in *x*.

### Data and estimation

In this section, we present the analyses that we carried out using the bivariate probit model described in the previous section. Our data came from the National Health Survey (NHS) of 2017 ([14]). This survey is representative of the Spanish population at the national and autonomous community levels. The main objective of this survey is to collect information about the main health indicators and the most important life condition factors of the population. The explanatory variables chosen in this model contain the characteristics that determine the probability of suffering gastrointestinal problems, such as age, marital status and education level, and variables that could affect one’s quality of life, such as suffering stress, being obese, doing exercises frequently or having limitations. The descriptive statistics for the main explanatory variables are reported in Table 1.

**Table 1:** Main statistics.

Variable	Definition	Mean	S.D.
**Individual Characteristics**			
Gastrointestinal Problems	Takes value 1 if the person has gastrointestinal problems	0.083	0.277
Depression	Takes a value of 1 if the person has depression	0.107	0.308
Anxiety	Takes a value of 1 if the person has anxiety	0.098	0.298
Age	Age in years	53.436	18.894
Married	Takes a value of 1 if is married	0.539	0.498
Male	Takes a value of 1 if is a man	0.458	0.498
High-level Studies	Takes a value of 1 if has high-level studies	0.181	0.385
Obese	Takes a value of 1 if is obese	0.169	0.375
Very stressed	Takes a value of 1 if declare to be very stressed	0.100	0.300
Exercise several times per week	Takes a value of 1 if practice exercise several times per week	0.113	0.317
Limitations	Takes a value of 1 if the person has limitations	0.671	0.051

Source: Own Elaboration from National Health Survey of 2017

The study population consisted of 23, 089 adults (aged 15 or more years). Table 1 shows the descriptive statistics for the major variables used in this research. In total, 45.8% of all subjects were men, 8.3% reported having any gastrointestinal problem, 10.7% reported having depression, and 9.8% reported having anxiety. Additionally, 16.9% were obese, 53.9% were married, and 11.3% reported engaging in exercise several times per week. More than half of the sample reported having some kind of limitation, and 10% were very stressed.

## Results

This study analyzes the associations between gastrointestinal pain and mental disorders such as depression or anxiety. In this section, the obtained results are presented. Tables 2 and 3 provide the results of each of the estimated methods. First, we estimate the probability of having depression and anxiety under the assumption that having gastrointestinal problems is an exogenous variable. Additionally, the marginal effects for these probit estimations can be seen in Table 2.

**Table 2:** Probability of having depression or anxiety and marginal effects.

Variables	Expected sign	Depression (Standard deviation in brackets)	Marginal Effect	Anxiety (Standard deviation in brackets)	Marginal Effect
Gastrointestinal Problems	+	0.600*** (0.082)	0.070	0.591*** 0.079	0.088
Age	+	0.016*** (0.002)	0.0012	0.006*** (0.002)	0.0006
Married	-	-0.313*** (0.047)	-0.024	-0.215*** (0.043)	-0.022
Male	-	-0.458*** (0.048)	-0.034	-0.381*** (0.044)	-0.037
High-level Studies	-	-0.248*** (0.056)	-0.016	-0.148** (0.049)	-0.013
Obese	+	0.055 (0.064)	0.004	0.032 (0.060)	0.003
Very stressed	+	0.411*** (0.062)	0.039	0.411*** (0.058)	0.052
Exercise several times per week	-	-0.242** (0.084)	-0.015	-0.144** (0.058)	-0.013
Limitations	+	0.671*** (0.051)	0.075	0.586*** (0.049)	0.079

Source: Own Elaboration from National Health Survey of 2017

**Table 3:** Bivariate probit of being depressed and having gastrointestinal problems.

Variables	Coefficient (Standard deviation in brackets)	P-value
**Depression**		
Age	0.012 (0.002)	0.000
Married	-0.330 (0.051)	0.000
Male	-0.455 (0.054)	0.000
High-level Studies	-0.317 (0.062)	0.000
Obese	0.017 (0.068)	0.800
Very stressed	0.401 (0.067)	0.000
Exercise several times per week	-0.239 (0.089)	0.008
Limitations	0.559 (0.054)	0.000
Constant	-1.685 (0.124)	0.000
**Gastrointestinal problems**		
Age	0.016 (0.002)	0.000
Married	0.002 (0.059)	0.934
Male	-0.236 (0.058)	0.000
High-level Studies	-0.111 (0.065)	0.091
Obese	0.024 (0.074)	0.743
Very stressed	0.094 (0.082)	0.252
Exercise several times per week	-0.247 (0.099)	0.013
Limitations	0.104 (0.066)	0.117
Smoking	-0.005 (0.066)	0.934
Sleeping Pills	0.178 (0.080)	0.027
Constant	-2.278 (0.147)	0.000
**athrho**	0.225 (0.048)	0.000

Source: Own Elaboration from National Health Survey of 2017

The first important fact we can observe is that all the expected signs coincide with the observed signs. In our case, we show that the probability of having depression and anxiety increases with age if the person has gastrointestinal problems, is very stressed or has any limitation. In contrast, being a man, married or engaging in exercises several times per week decreases the probability of having depression or anxiety. According to this first estimate, having gastrointestinal problems increases the chance of having depression in 7% and the probability of having anxiety problems in 8.8% of the sample. In the next tables (Tables 3 and 4), the bivariate probit models are reported. These estimation results confirm the high correlation between the two decisions, which is corroborated using the Wald test of the correlation coefficient of the error terms (*rho*). In this case, we cannot reject the null hypothesis that the unobserved factors affecting the probability of having depression and the probability of having gastrointestinal problems are uncorrelated. The correlation coefficient is significantly different from zero; thus, we cannot proceed by estimating a separate probit model. The bivariate probit model is an adequate procedure for modeling the anxiety and depression related to having gastrointestinal problems because both mechanisms are interrelated and cannot be estimated independently.

**Table 4:** Bivariate probit of having anxiety and having gastrointestinal problems.

Variables	Coefficient (Standard deviation in brackets)	P-value
**Depression**		
Age	0.0044 (0.002)	0.052
Married	-0.270 (0.048)	0.000
Male	-0.320 (0.049)	0.000
High-level Studies	-0.192 (0.055)	0.001
Obese	-0.008 (0.063)	0.893
Very stressed	0.375 (0.064)	0.000
Exercise several times per week	-0.250 (0.080)	0.002
Limitations	0.425 (0.052)	0.000
Constant	-1.257 (0.112)	0.000
**Gastrointestinal problems**		
Age	0.016 (0.002)	0.000
Married	0.001 (0.059)	0.975
Male	-0.237 (0.058)	0.000
High-level Studies	-0.112 (0.065)	0.087
Obese	0.022 (0.074)	0.766
Very stressed	0.091 (0.082)	0.270
Exercise several times per week	-0.247 (0.099)	0.013
Limitations	0.100 (0.066)	0.132
Smoking	-0.004 (0.064)	0.943
Sleeping Pills	0.188 (0.081)	0.021
Constant	-2.283 (0.148)	0.000
**athrho**	0.188 (0.047)	0.000

Source: Own Elaboration from National Health Survey of 2017

As we can see in Tables 3 and 4, females, older individuals, very stressed people or those with any limitation are more likely to suffer depression or anxiety problems than are the rest of the sample. Additionally, married people, those with higher education levels and those who practice exercise frequently are less likely to suffer the examined mental problems.

It can be observed in Table 5 that the estimated effect is positive, which is consistent with the hypothesis that having gastrointestinal problems is associated with a higher probability of having depression or anxiety. The estimated effect of having gastrointestinal problems on depression is an increase of 8.2% for the mean difference, while holding the rest of the factors constant, and it is an increase of 8% in the case of anxiety problems. Additionally, 16.4% of people with gastrointestinal problems are estimated to have depression, while only 8.3% of people without gastrointestinal problems are estimated to have depression. Moreover, 17.9% of people with gastrointestinal problems are estimated to have anxiety problems, while only 9.9% of people without gastrointestinal problems are estimated to have anxiety.

**Table 5:** Predicted probabilities.

Variable	Mean	Std. Dev.
Estimated Probability of having depression with gastrointestinal problems	0.165	0.109
Estimated Probability of having depression without gastrointestinal problems	0.083	0.073
Difference	0.082	0.037
Estimated Probability of having anxiety with gastrointestinal problems	0.179	0.087
Estimated Probability of having anxiety without gastrointestinal problems	0.099	0.062
Difference	0.080	0.02

Source: Own Elaboration from National Health Survey of 2017

When age increases by 1 year, the probability of someone having depression or anxiety without having gastrointestinal problems increases by 0.1%, holding all the explanatory variables constant. When age increases by 1 year, the probability of someone having both depression and gastrointestinal problems increases by 0.03% (see Table 6). If the person is married, then his or her probability of having both depression and a gastrointestinal problem decreases by 0.3%, while his or her probability of having both anxiety and a gastrointestinal problems decreases by 4.6% with respect to others who are married, holding the factors fixed. Moreover, if the person is a man, then his probability of having both depression and a gastrointestinal disease decreases by approximately 0.8% in comparison to a woman. For males, the probability of having anxiety from a gastrointestinal disease decreases by approximately 5.1% compared to their female counterparts. In the case of those with a higher-level education, the probability of having both depression and a gastrointestinal problem decreases by 0.5%. Additionally, for more educated people, the probability of having both anxiety and a gastrointestinal problem decreases by 2.9%. For very stressed individuals, the probability of having depression with a gastrointestinal problem increases by 0.5%, while the probability of having anxiety with a gastrointestinal problem increases by 7.4%. If the person engages in some exercise, the probability of him or her having depression without having gastrointestinal problems decreases by approximately 6.5%, ceteris paribus (and decreases by 0.6% if he or she has gastrointestinal problems). In this sense, if the person engages in some exercise, then his or her probability of having anxiety without having gastrointestinal problems decreases by approximately 1.8%, ceteris paribus (and decreases by 3.6% if he or she has gastrointestinal problems).

**Table 6:** Marginal effects.

Variables	Marginal effects(p00)	Marginal effects(p10)	Marginal effects(p01)	Marginal effects(p11)
**Depression**				
Age	-0.0029	0.0012	0.0013	0.0003
Married	0.039	-0.039	0.004	-0.003
Male	0.075	-0.051	-0.016	-0.008
High-level Studies	0.048	-0.036	-0.006	-0.005
Obese	-0.002	-0.000	0.002	0.000
Very stressed	-0.056	0.046	0.003	0.005
Exercise severaltimes per week	0.051	-0.065	-0.019	-0.006
Limitations	-0.076	0.065	0.002	0.008
Smoking	-0.016	-0.002	0.016	0.002
Sleeping Pills	0.000	0.000	-0.000	-0.000
**Anxiety**				
Age	-0.002	0.001	0.000	0.000
Married	0.040	0.003	-0.048	-0.046
Male	0.069	-0.017	-0.044	-0.051
High-level Studies	0.039	-0.007	-0.027	-0.029
Obese	-0.000	0.002	-0.001	-0.001
Very stressed	-0.065	0.003	0.055	0.074
Exercise several times per week	0.059	-0.018	-0.034	-0.036
Limitations	-0.073	0.003	0.063	0.082
Smoking	-0.017	-0.000	0.000	0.000
Sleeping Pills	0.000	0.000	-0.000	-0.000

Source: Own Elaboration from National Health Survey of 2017

## Discussion

Some of the most important neuropsychiatric disorders, including schizophrenia and bipolar disorder, are still lack of deep insights of the etiology and biological mechanisms ([15–20]). In this sense, few studies investigate that immune activation may be related with the etiopathogenesis of these mental diseases. There exist some studies with animals which indicate that the mucosal microbiome can be related with the behavior by altering the immune system. Then, it is possible to think that there exists an association between the microbiome and human mental diseases. Unfortunately, the investigation in this issue is still scarce. There is, so far, very little literature in these issues but the existent about the oropharyngeal microbiota in schizophrenia found significant differences between the treated group and the controls. Additively, other investigations have found that gastrointestinal inflammation in population with schizophrenia or bipolar disorders basing the results on measures of microbial translocation. It has been also found a relation between taking antibiotics and psychiatric disorders, perhaps due to alterations in the microbiome [21]. Severance et al. [22] investigated the relationship between gastrointestinal disturbances and mental diseases, concretely, the schizophrenia. They conclude that some risk factors for schizophrenia (stress, some intolerances, inflammation, sleep, cellular barrier defects) are some biological pathways that also contribute to alter the gut microbiota.

Scientifically, there exists some evidences associating gut microbiota to both gastrointestinal and non-gastrointestinal diseases. According to that, a strand of “new” literature is in fact emerging recent literature is emerging focusing on the variation of the microbiome and the effect that it has on central nervous system diseases. Some experimental researches corroborate this such as Zheng et al. [23] found a reduction in alpha diversity among the severe combined immunodeficiency (SCI) and Non obese diabetic/severe combined immunodeficiency (NO/SCI) mouse groups. This finding suggests that this is a consequence of microbial dysbiosis, which may predispose the mice to inflammatory complications and infections. Then, these authors demonstrated that a decrease in alpha diversity and a clear structural separation exist in the microbiota of immunodeficient mice. Dysbiosis and inflammation of the gut have been linked to causing several mental illnesses including anxiety and depression, which are the objective of this article. Then, this empirical evidence supports the hypothesis that gastrointestinal problems are associated with mental illnesses such as depression or anxiety.

According to [24] the pathogenesis of inflammatory bowel disease is believed to involve an aberrant immune response to intestinal microbiota in genetically susceptible individuals. In this sense the research in Epigenomics, in multiple directions, is becoming a clue into the pathogenesis of some important diseases. It is important to highlight that several RNA modifications are known to decorate RNAs and impact its structure and function. One such recently discovered modification is acetylation of RNA i.e. N4-acetylcytidine (ac4C) chemical modification. N4-acetylcytidine is an ancient and evolutionarily conserved modification, which maps to a wide spectrum of RNAs from archaea bacteria to humans. This modification results in a variety of functional outcomes which impact normal development and disease [25, 26].

However, there are some limitations associated with this study. We acknowledge that both depression and anxiety have different levels of intensity; thus, the use of scale variables instead of binary variables is recommended, but the data used herein do not allow this disaggregation. Additionally, the majority of the variables are self-reported, which could lead to some bias in our results because the individuals are reporting on their own lives and they could have incentives to not report realistically. Finally, our study is cross-sectional; thus, effects derived from the passing of time are not considered. In this way, it would be interesting as a future line of research to analyze whether the impact of gastrointestinal problems and mental disorders becomes stronger over time. Meanwhile, in our cross-sectional study, the observed effects of gastrointestinal disturbances on the onset of depression or anxiety should be further explored to determine the causality by using Mendelian randomization analysis based on systems-level genomic data and environmental exposure mensurements ([8, 27–29]) Even considering these limitations, our research has several strengths. Few studies have previously empirically analyzed the association between gastrointestinal pain and depression or anxiety problems. Our study allows the joint modeling of both outcomes due to their significant correlation. There are some important findings in our study: the estimated effect of gastrointestinal problems on mental health is positive, which is consistent with the initial hypothesis. The estimated effect of having gastrointestinal problems on depression is an increase of 8.2% for the mean difference, while holding the rest of the factor’s constant, and it is an increase of 8% in the case of anxiety problems. Moreover, our results indicate that the probability of having both depression and a gastrointestinal problem is higher with age, for more stressed people. On the other hand, this probability decreases for married people, men, more educated individuals and if the person engages in some exercise. Additionally, few recent studies have investigated the relationship between gastrointestinal problems and mental health considering some socioeconomic variables as controls.

## Conclusions

This study assessed the impact of having gastrointestinal issues on the onset of depression or anxiety in the Spanish population. Moreover, in this research, we showed the application of a bivariate probit model to deal simultaneously with these two binary outcomes. We tested whether the simultaneous joint treatment of both outcomes was the best alternative due to the endogenous process. In summary, our empirical results suggest that depression and anxiety problems in patients with gastrointestinal symptoms require special attention and that it is necessary to develop public health strategies that consist of early attention, special care and psychological interventions. In this context, it is important to avoid barriers to accessing specialist mental health care for people with other associated diagnoses, as is the case for gastrointestinal disorders, according to our results. Finally, our policy implications recommend the provisions of preventive mental health care and assistance with managing several chronic illnesses that can worsen one’s mental disorders.
